# Antioxidant Properties and Fatty Acid Profile of Cretan Extra Virgin Bioolive Oils: A Pilot Study

**DOI:** 10.1155/2021/5554002

**Published:** 2021-03-25

**Authors:** Dariusz Nowak, Michał Gośliński, Cezary Popławski

**Affiliations:** Department of Nutrition and Dietetics, Faculty of Health Sciences, Ludwik Rydygier Collegium Medicum in Bydgoszcz, Nicolaus Copernicus University in Toruń, Dębowa 3, 85-626 Bydgoszcz, Poland

## Abstract

Olive oil is considered a valuable ingredient of human diet. It is a good source of mono- and polyunsaturated fatty acids, as well as other bioactive compounds, especially polyphenols. The composition of olive oil depends mainly on the variety of plant, cultivation practices, and manufacturing conditions. Traditional processing methods may ensure better quality and health benefits. Therefore, the aim of the study was the evaluation of antioxidant properties and fatty acid profile of Cretan extra virgin bioolive oils. These ones were compared with commercial Spanish, Italian, and Greek extra virgin olive oils. Obtained results showed that sample Cretan 1 had about 15% higher antioxidant capacity and about 60% higher total polyphenol content than commercial counterparts. This one had also a favorable profile of fatty acids, especially 20% more linoleic acid. We concluded that traditional production methods, using millstones, cold pressing, and without centrifugation and filtration ensure better olive oil quality and related health benefits.

## 1. Introduction

Olive oil is a popular source of fats in the human diet, especially in the Mediterranean diet. In general, olive oil is characterized by high amounts of monounsaturated fatty acid (MUFA) and contains minor components with biological properties (e.g., phenolic compounds, pigments, squalene, sitosterols, and triterpenes) [1]. A specific composition of olive oil fatty acids and other bioactive compounds, such as polyphenols, has been proven to be protective against the development of cardiovascular diseases [2–5]. The content of these components depends on the cultivar, climate, harvesting time, and the manufacturing conditions [1, 6].

According to the European Union legislation, olive oil is classified into categories reflecting its quality and organoleptic properties, namely, extra virgin olive oil (EVOO), virgin olive oil (VOO), lampante virgin olive oil (LVOO), refined olive oil (ROO), and also olive oil (OO) [7, 8]. EVOO produced by mechanically pressing the olives is considered the best quality and possesses the best composition of bioactive compounds, which affect its health benefits [9, 10]. These olive oils contain an array of phenolic antioxidants, from three major chemical classes: the simple phenolics (tyrosol and hydroxytyrosol), the secoiridoids (oleuropein aglycon and oleocanthal), and lignans [11–13] and phenolic acids (vanillic, chlorogenic, gallic, caffeic, *p*-coumaric, and ferulic) [13].

Greece is ranked third after Spain and Italy in virgin olive oil production. In Greece and Italy, the extra virgin olive oil is consumed in majority, whereas in Spain, this represents less than half. The dominant olive cultivar in Greece is the cv. Koroneiki, especially on Crete. This gives oils of medium to high content of phenolic compounds. Greek extra virgin bioolive oils, produced mainly with traditional, nonintensive cultivation practices, are mostly of exceptional quality [14, 15]. Recent study shows that Greek EVOO had a high content of oleocanthal and oleacein and their derivatives [12]. These compounds are considered key oxidation inhibitors. It is worthy to note that oleacein has been declared a more potent antioxidant than hydroxytyrosol [16, 17]. A study showed that the specific phenolic content within EVOO can affect human health [18, 19]. Literature data for Greek, especially Cretan extra virgin olive oils, are not as popular as those for Spanish or Italian ones. Therefore, the aim of the study was the evaluation of antioxidant properties and the fatty acid profile of Cretan extra virgin bioolive oils. These ones were compared with commercial Spanish, Italian, and Greek extra virgin olive oils.

## 2. Materials and Methods

### 2.1. Materials

The analysis comprised five extra virgin olive oils. Spanish, Italian, and Greek samples were purchased at a local health food store and declared as extra virgin. Cretan extra virgin bioolive oils were produced from cv. Koroneiki (Chania region, northwest Crete) and have been certificated by BIO HELLAS no. B-515504 (Cretan 1) and no. B-515573 (Cretan 2). Moreover, sample Cretan 1 comes from two hundred years of organic cultivation (i.e., manual harvesting, without artificial irrigation), and this olive oil was produced by a traditional method using millstones, cold pressing, and without centrifugation and filtration. All the analyzed olive oils were produced between April and May 2018 (bottled into 250 mL) and had at least one-year best-before date.

### 2.2. Methods

For the spectrophotometric assays, 1 ± 0.01 g of olive oil was extracted with 9.3 mL n-hexane and homogenized using a vortex mixer for 30 sec. For each olive oil, five parallel samples in three replicates were prepared.

### 2.3. DPPH Assay

The antioxidant capacity of the olive oils was determined in extracts (1 g of olive oil and 9.3 mL n-hexane) by a standard DPPH method using 0.1 mM methanol solution of a 1,1-diphenyl-2-picrylhydrazyl (DPPH, Sigma-Aldrich) [20]. This method is widely used to test the antioxidant capacity of foods, including olive oils [21]. The absorbance was measured on a Hitachi U-1900 spectrophotometer at 517 nm after 30 min of incubation in the dark at room temperature. For each olive oil, five parallel samples in three replicates were analyzed, from which the mean value was calculated. The antioxidant capacity was expressed as milligrams of Trolox per 1 liter of olive oil (mg Tx/L).

### 2.4. ABTS Assay

The antioxidant capacity of the olive oils was determined by Re et al.'s [22] method using 2,2′-azinobis-(3-ethyl-benzothiazoline-6-sulfonic acid) diammonium salt (ABTS, Sigma-Aldrich). The absorbance was measured on a Hitachi U-1900 spectrophotometer at 734 nm after 6 min of incubation in the dark at room temperature. For each olive oil, five parallel samples in three replicates were analyzed, from which the mean value was calculated. The antioxidant capacity was expressed as milligrams of Trolox per 1 liter of olive oil (mg Tx/L).

### 2.5. Folin-Ciocalteu Assay

The total polyphenols content (TP) of the samples was determined by the Folin-Ciocalteu assay [23]. The absorbance was measured on a Hitachi U-1900 spectrophotometer at 765 nm after 30 min incubation in the dark at room temperature. The results were expressed as milligrams of gallic acid equivalents per 1 liter of olive oil (mg GAE/L).

### 2.6. Determination of Fatty Acid Profile

The fatty acid profile was analyzed according to the European Union Commission Regulation [24] using a Hewlett-Packard 6890 gas chromatograph, equipped with a flame-ionization detector (FID) and a SP-2560 fused silica capillary column (100 m × 0.25 mm; 0.20 *μ*m film thickness). The injector and detector temperatures were set at 220°C and 240°C, respectively. Helium was used as a carrier gas with a flow rate of 1 mL min^−1^. The analysis was performed at the following temperature program: 140°C held for 5 min, then increased at rate of 4°C/min to 240°C, and held for the subsequent 20 min. The total run time was approximately 50 min. Individual fatty acids were identified by comparing their retention times with standards and quantified as a percentage of the total fatty acids.

### 2.7. Statistical Analysis

The results were statistically analyzed by calculating the mean and standard deviation. The interpretation of the results was performed with MS Excel 2010 Analysis ToolPak software, with one-way analysis of variance (ANOVA) using Tukey's as a posttest: different letters in the same row indicate statistical significance (at *p* < 0.05).

## 3. Results and Discussion

### 3.1. Antioxidant Properties

Obtained results showed that among all the analyzed olive oils, the significantly highest antioxidant capacity, both the DPPH and ABTS assays, was determined in sample Cretan 1, i.e., 348 ± 5.0 and 623 ± 7.0 mg Tx/L, respectively (Table 1). Olive oil Cretan 1 had also the highest total polyphenol content (658 ± 40 mg GAE/L). Other extra virgin olive oils were characterized by TP about 400 mg GAE/L, with no significant differences between them (*p* < 0.05). The sample Cretan 2 was similar in antioxidant capacity to commercial Greek and Italian olive oils, but their values were slightly lower than the Spanish one.

Other researchers reported a great variability in TP ranged from 50 to 1000 mg/kg (usually 100-300 mg/kg) in olive oils of different origin, i.e., Greek, Italian, Spanish, Israeli, and Turkish [14, 25, 26]. Total polyphenol content has been repeatedly proved to be a marker for olive oil stability, which is also related to characteristic taste. Moreover, TP was considered a parameter which categorized olive oils as low (50–200 mg GAE/kg), medium (200–500 mg GAE/kg), and high (500–1000 mg GAE/kg) [14]. In addition, literature data stated that cv. Koroneiki gives olive oils of higher TP content, in general [27]. In another study, fifty-five mono- or multivarietal extra virgin olive oils from Italy, Spain, France, Turkey, Greece, Portugal, Australia, the USA, and South Africa were analyzed. The oil samples produced from Italian cv. Coratina possessed the highest amount of polyphenols and antioxidant capacity, whilst the sample produced from French cv. Cayon contained the lowest amount. Among the analyzed olive oils was also some Cretan of cv. Koroneiki, of which its antioxidant properties were average in relation to the others [28]. Moreover, Sicari [29] determined the antioxidant capacity, TP, and structure of polyphenolic compounds in three different Italian extra virgin olive oils from the province of Reggio Calabria. The analyzed samples had a high content of polyphenols ranged 370-530 mg GAE/kg. Moreover, in this study, a positive correlation was observed between the antioxidant activity (determined by DPPH and ABTS assay) and the concentration of total polyphenols [29]. Our results also confirmed positive correlation between the DPPH and ABTS methods (*r* = 0.902). Furthermore, the ABTS assay resulted in a much higher value of antioxidant capacity, which was also confirmed in other studies [30, 31]. De Bruno et al. [31] reported that it could be due to the different composition of analyzed samples containing hydrophilic and lipophilic antioxidant compounds. The ABTS assay is more applicable to both hydrophilic and lipophilic antioxidant systems, whereas DPPH assay is more related to hydrophobic system response [32]. In addition, our results showed a higher correlation between ABTS and TP (*r* = 0.912) than between DPPH and TP (*r* = 0.738).

On the other hand, Condelli et al. [33] reported that the differences in antioxidant capacity may depend on the composition and profile of phenolic compounds, rather than total polyphenol content. This study was conducted on 75 Italian commercial extra virgin olive oils. Finally, Jimenez-Lopez et al. [34] pointed out various factors that affect the quality of EVOO and its bioactive compounds. Bruno et al. [31] concluded that harvesting time and climate conditions influence the phenol composition as ratios of phenol compounds and their total amount.

### 3.2. Fatty Acid Profile

The appropriate profile of fatty acids determines the quality and health benefits of olive oil. The analyses showed that the tested olives had a typical content of oleic acid C18 : 1 (above 72%). Particularly important for health are polyunsaturated fatty acids (PUFA). Sample Cretan 1 had about 20% more linoleic acid C18 : 2, whereas the content of *α*-linolenic acid C18 : 3 did not differ significant between all the samples (Table 2).

The mean values of fatty acid profile found in the present study were the within limits established by the International Olive Oil Council for purity criteria of olive oils [35]. Stefanoudaki et al. [15] reported that Greek olive oils of cv. Koroneiki were characterized by a higher concentration of oleic acid C18 : 1 (74.7–79.9%), but the concentration of linoleic acid C18 : 2 and *α*-linolenic acid C18 : 3 was slightly lower (ca. 5.0–7.0% and 0.55–0.76%, respectively). Mikrou et al. [36] studied 68 monovarietal EVOOs, originating from three regions of Greece and two local cultivars (Koroneiki and Kolovi), and reported similar concentration oleic, linoleic, and *α*-linolenic acid. The exception was the cultivar Kolovi, which had a higher linoleic acid content of 11.63% [36]. Other studies showed that Italian and Spanish olive oils had even lower values of C18 : 3, i.e., 0.49–0.54% [37] and 0.48% [38], respectively. These values were much lower than obtained for commercial olive oils in our studies, whereas Morello et al. [39] reported similar to our values of C18 : 3. Taking into account the content of PUFA, the tested samples present the upper range of these compounds, which could be found in literature data. Furthermore, Stefanoudaki et al. [15] stated that lower concentration of oleic acid resulted in a higher concentration of heptadecanoic C17 : 0 and linoleic C18 : 2 acids, which was also confirmed in our research. Therefore, Kosma et al. [40] concluded that variations in the fatty acid composition may be owed to factors such as cultivar and other factors for example climatic conditions and geographical origin. The reported statement for Greek oils has been also confirmed in different studies on Italian olive oils. Moreover, Piscopo et al. [41] demonstrated the effect of olive cultivar and the environmental influences on the fatty acid composition of monovarietal olive oils, considering also the quality of a same cultivar in different areas.

We are aware that our study has some limitations. In the next studies, the profile of polyphenolic compounds and other valuable parameters such as tocopherol, squalene, oleocanthal, and oleacein should be examined.

## 4. Conclusions

The results of our research showed that the sample Cretan 1 had about 15% higher antioxidant capacity and about 60% higher total polyphenol content than Spanish, Italian, and Greek extra virgin olive oils. These olive oil had also a favorable composition of fatty acids, especially linoleic and *α*-linolenic acid. The sample Cretan 2 did not differ significant from the commercial counterparts.

In conclusion, the antioxidant properties depend on the manufacturing conditions. Oils from olives grown on organic farms (manual harvesting, without artificial irrigation) and produced with traditional methods, i.e., using millstones, cold pressing, and without centrifugation and filtration, had higher antioxidant properties and favorable profile of fatty acids.

## Figures and Tables

**Table 1:** Antioxidant capacity and total polyphenols of extra virgin olive oils.

	Spanish	Italian	Greek	Cretan 1	Cretan 2
DPPH (mg Tx/L)	324 ± 5.0^b^	313 ± 3.0^c^	290 ± 4.0^d^	348 ± 5.0^a^	282 ± 1.0^d^
ABTS (mg Tx/L)	552 ± 7.0^b^	513 ± 5.0^c^	501 ± 5.0^c^	623 ± 7.0^a^	517 ± 6.0^c^
TP (mg GAE/L)	409 ± 36^b^	393 ± 62^b^	403 ± 86^b^	658 ± 40^a^	423 ± 14^b^

Data are mean ± SD (*n* = 5). Different letters in the same row indicate statistical significance at *p* < 0.05; Tx: Trolox equivalents; GAE: gallic acid equivalents.

**Table 2:** Fatty acid profile of extra virgin olive oils (%).

	Spanish	Italian	Greek	Cretan 1	Cretan 2
Caprylic acid C8 : 0	nd	nd	nd	nd	nd
Capric acid C10 : 0	nd	nd	nd	nd	nd
Lauric acid C12 : 0	0.03 ± 0.04	nd	nd	nd	nd
Tridecanoic acid C13 : 0	nd	nd	nd	nd	nd
Myristic C14 : 0	nd	nd	nd	nd	nd
Myristoleic acid C14 : 1	nd	nd	nd	nd	nd
Pentadecanoic acid C15 : 0	nd	nd	nd	nd	nd
Palmitic acid C16:0	13.08 ± 0.55	12.98 ± 0.08	12.93 ± 0.16	13.20 ± 0.25	12.94 ± 0.14
Palmitoleic acid C16 : 1	1.05 ± 0.23^a^	0.67 ± 0.72^ab^	0.53 ± 0.57^b^	0.12 ± 0.02^c^	0.12 ± 0.01^c^
Heptadecanoic acid C17 : 0	0.15 ± 0.04^b^	0.63 ± 0.75^a^	0.47 ± 0.58^a^	0.84 ± 0.02^a^	0.77 ± 0.01^a^
Stearic acid C18 : 0	3.25 ± 0.02^a^	2.86 ± 0.07^b^	2.76 ± 0.06^b^	2.87 ± 0.01^b^	2.91 ± 0.03^b^
Elaidic acid C18 : 1 n9t	nd	nd	nd	nd	nd
Oleic acid C18 : 1 n9c	72.77 ± 0.14^c^	73.74 ± 0.16^b^	75.29 ± 0.01^a^	72.49 ± 1.29^bc^	74.18 ± 0.10^b^
Linoleic acid C18:2	8.13 ± 0.04^b^	7.60 ± 0.10^c^	6.44 ± 0.06^d^	8.94 ± 0.62^a^	7.56 ± 0.03^c^
*α*-Linolenic acid C18:3	0.76 ± 0.04	0.72 ± 0.06	0.74 ± 0.01	0.73 ± 0.01	0.72 ± 0.01
Arachidic acid C20 : 0	0.39 ± 0.01^c^	0.48 ± 0.11^a^	0.41 ± 0.01^bc^	0.41 ± 0.03^bc^	0.44 ± 0.01^b^
11-Eicosenoic acid C20 : 1	0.27 ± 0.02	0.27 ± 0.01	0.30 ± 0.04	0.26 ± 0.01	0.28 ± 0.01
Behenic acid C22 : 0	0.11 ± 0.01^b^	0.06 ± 0.08^c^	0.13 ± 0.01^ab^	0.14 ± 0.01^a^	0.07 ± 0.11^c^
Erucic acid C22 : 1	nd	nd	nd	nd	nd
MUFA (%)	74.09^b^	74.68^b^	76.12^a^	72.87^b^	74.58^b^
PUFA (%)	8.89^b^	8.32^b^	7.18^c^	9.67^a^	8.28^b^
SFA (%)	17.01	17.00	16.7	17.46	17.14

Data are mean ± SD (*n* = 4). Different letters in the same row indicate statistical significance at *p* < 0.05; nd: not detected; MUFA: monounsaturated fatty acids; PUFA: polyunsaturated fatty acids; SFA: saturated fatty acids.

## Data Availability

The results are in the article and in the corresponding author.
